# Endogenous n-3 Fatty Acids Alleviate Carbon-Tetrachloride-Induced Acute Liver Injury in* Fat-1* Transgenic Mice

**DOI:** 10.1155/2016/7962948

**Published:** 2016-11-06

**Authors:** Ruibing Feng, Meng Wang, Chunyan Yan, Peng Li, Meiwan Chen, Chengwei He, Jian-Bo Wan

**Affiliations:** ^1^State Key Laboratory of Quality Research in Chinese Medicine, Institute of Chinese Medical Sciences, University of Macau, Macau; ^2^College of Pharmacy, Guangdong Pharmaceutical University, Guangzhou 510006, China

## Abstract

n-3 polyunsaturated fatty acids (PUFAs) are beneficial for numerous models of liver diseases. The probable protective effects of n-3 PUFA against carbon-tetrachloride- (CCl_4_-) induced acute liver injury were evaluated in a* fat-1* transgenic mouse that synthesizes endogenous n-3 from n-6 PUFA.* Fat-1* mice and their WT littermates were fed a modified AIN93 diet containing 10% corn oil and were injected intraperitoneally with a single dose of CCl_4_ or vehicle. CCl_4_ challenge caused severe liver injury in WT mice, as indicated by serum parameters and histopathological changes, which were remarkably ameliorated in* fat-1 *mice. Endogenous n-3 PUFA decreased the elevation of oxidative stress induced by CCl_4_ challenge, which might be attributed to the activation of Nrf2/keap1 pathway. Additionally, endogenous n-3 PUFA reduces hepatocyte apoptosis via suppressing MAPK pathway. These findings indicate that n-3 PUFA has potent protective effects against acute liver injury induced by CCl_4_ in mice, suggesting that n-3 PUFA can be used for the prevention and treatment of liver injury.

## 1. Introduction

Liver is a vital organ that has extensive synthetic, metabolic, and detoxifying functions [1]. This tissue is also a main target that is subject to acute or chronic injury induced by a variety of drugs or xenobiotics, such as alcohol, heavy metals, and carbon tetrachloride (CCl_4_) [2]. CCl_4_, an analogue of human hepatotoxin, has been widely used in* in vitro* and* in vivo* models to induce liver injury [3]. CCl_4_ is oxidized by cytochrome P4502E1 (CYP2E1) in the liver to generate the highly reactive species, such as trichloromethyl (^∙^CCl_3_) and peroxy trichloromethyl (^∙^OOCCl_3_) radicals [4], which can trigger oxidative stress, lipid peroxidation, and hepatocyte apoptosis, leading ultimately to hepatotoxicity [5].

Oxidative stress is mainly responsible for the pathogenesis of CCl_4_-induced liver injury, which can disturb the redox homeostasis and elevate the excessive production of reactive oxygen species (ROS) [6]. Antioxidant defense system, including nonenzymatic antioxidants and enzymatic antioxidants, contributes to protecting the liver against oxidative stress in living organisms. The expressions of these antioxidative enzymes are regulated by a redox-sensitive transcription factor, nuclear factor-erythroid 2-related factor-2 (Nrf2), and its downstream proteins, including heme oxygenase-1 (HO-1), glutamate cysteine ligase (GCL), and NAD(P)H:quinone oxidoreductase-1 (NQO1) [7, 8]. Oxidative stress also elevates cytochrome C in the cytoplasm which is released from the mitochondria, which induces the activation of caspase cascades, eventually leading to hepatocyte apoptosis.

Growing evidence indicates that n-3 polyunsaturated fatty acids (PUFAs), mainly *α*-linolenic acid (ALA), eicosapentaenoic acid (EPA), and docosahexaenoic acid (DHA), exhibit profoundly therapeutic efficacy in several models of liver disease, including nonalcoholic liver disease [9], parenteral nutrition-associated liver disease [10], alcohol-induced liver injury [11], hepatic steatosis [12, 13], and D-galactosamine/lipopolysaccharide-induced hepatitis [14]. However, the impacts of n-3 PUFA on CCl_4_-induced liver injury have not been sufficiently addressed. The* fat-1* transgenic mouse was genetically modified to express a* fat-1* gene that encodes n-3 PUFA desaturase [15, 16]. This enzyme can endogenously convert n-6 PUFA to n-3 PUFA in mammals, leading to higher n-3 PUFA level in tissues from* fat-1 *mice, compared to the wild-type (WT) littermates when fed the same diet rich in n-6 PUFA. Thus,* fat-1* mice are a well-established animal model to investigate the role of n-3 PUFA in CCl_4_-induced liver injury. Therefore, the aims of current study are to evaluate the probable effects of n-3 PUFA against CCl_4_-induced acute liver injury and to elucidate the potential molecular mechanisms underlying this action.

## 2. Materials and Methods

### 2.1. Animals and Treatments

*Fat-1* transgenic mice with a genetic background of C57BL/6 were provided by Dr. Jing X. Kang's lab at Massachusetts General Hospital (Boston, MA, USA). Male heterozygous* fat-1* mice were crossed with C57BL/6 female mice to yield heterozygous* fat-1 *and WT offspring. The* fat-1 *phenotype of each offspring was identified by the analysis of total lipids from mouse tail by using gas chromatography-mass spectrometry (GC-MS). The female* fat-1*-positive and WT littermates were maintained in a specific pathogen-free room at the Experimental Animal Center, Guangdong Pharmaceutical University. Mice were fed an n-6 PUFA-rich but n-3 PUFA-deficient diet (a modified AIN93 containing 10% corn oil), which contains 20% protein, 58% carbohydrate, and 22% fat (TROPHIC Animal Feed High-Tech Co., Ltd., Nantong, China). Mice (10–12 weeks old) were divided into three groups (*n* = 10), that is, WT control, WT/CCl_4_, and* fat-1*/CCl_4_ groups. The mice in CCl_4_ challenge groups were injected intraperitoneally with 10 mL/kg CCl_4_ (0.2%, dissolved in olive oil) [17], while WT controls received an equal volume olive oil (i.p.). After 24 h of CCl_4_ or vehicle treatment, all mice were anesthetized by an injection (i.p., 100 mg/kg) with sodium pentobarbital. Blood samples and liver tissues were immediately collected. The animal experimental protocols (ICMS-AEC-2015-028) were performed according to the Guide to Animal Use and Care of the University of Macau and were approved by the ethics committee.

### 2.2. Analysis of Fatty Acid Composition in the Liver

Fatty acid profile was analyzed according to a simplified method by using GC-MS, as described previously [18, 19]. In brief, approximately 10 mg of liver tissue was ground in liquid nitrogen and methylated with 1.5 mL of 14% boron trifluoride-methanol reagent (Sigma-Aldrich) and 1.5 mL of hexane at 100°C for 1 h. After cooling, fatty acid methyl esters (FAME) were extracted in upper hexane layer. GC-MS analysis was conducted on a Thermo Fisher Scientific ISQ Series Single Quadrupole GC-MS system equipped with a TriPlus RSH™ autosampler (Thermo Fisher, Waltham, MA, USA). Separation of FAME was achieved on an Omegawax™ 250 fused silica capillary column (30 m × 0.25 mm i.d., 0.25 *μ*m film thickness, Supelco, Bellefonte, PA). The optimum oven temperature program was as follows: it was initially set at 180°C for 3 min, ramped to 206°C at 2°C/min, held at 206°C for 25 min, ramped to 240°C at 10°C/min, and held at 240°C for 5 min. Peaks in the chromatogram were identified by comparing their retention times and mass spectrums with GLC-461 reference standard (Nu-Chek Prep, Elysian, MN, USA) containing 32 FAME.

### 2.3. Measurement of Serum Aminotransferase Levels

Activities of serum aspartate aminotransferase (AST) and alanine aminotransferase (ALT) were colorimetrically examined by using their commercial kits (Nanjing Jiancheng Bioengineering Institute, Nanjing, China).

### 2.4. Determination of Oxidative Stress Parameters in the Liver

Partial liver tissues were weighed and homogenized with cold radioimmunoprecipitation assay (RIPA) buffer (Beyotime Institute of Biotechnology, Nanjing, China) to prepare 10% liver homogenate. After centrifugation, the supernatant was subjected to measure the levels of malondialdehyde (MDA), reduced glutathione (GSH), and oxidized glutathione (GSSG) and the activities of catalase (CAT), glutathione peroxidase (GSH-Px), glutathione reductase (GR), and superoxide dismutase (SOD) in the liver, by the corresponding kits (Nanjing Jiancheng Bioengineering Institute). Total proteins in the homogenate were quantified using Pierce™ BCA Kit (Thermo Fisher). Values were normalized against hepatic total protein content.

### 2.5. Histopathological Analysis

Histopathological changes of the liver were observed by hematoxylin and eosin (H&E) staining [20]. The liver tissue from the same lobe in each mouse was fixed in 10% formalin overnight, dehydrated in alcohol with different concentrations, and embedded in paraffin. The liver sections (5 *μ*m thickness) were stained with H&E using a standard protocol. The histopathological changes of each mouse were examined and photographed by an Olympus CX-31 light microscope (Olympus Corp., Tokyo, Japan).

### 2.6. TUNEL Assay

To evaluate apoptotic cells in the liver tissue, a terminal deoxynucleotidyl transferase-mediated deoxyuridine 5-triphosphate (dUTP) nick end labeling (TUNEL) assay was conducted by using ApopTag® Plus In Situ Apoptosis Fluorescein Detection Kit (S7111, EMD Millipore Corporation, Billerica, MA, USA). In brief, liver cryostat section (8 *μ*M) was fixed in 1% paraformaldehyde and washed with PBS three times. Then, the section was incubated with green fluorescein labeled dUTP solution at 37°C for 1 h. The section was counterstained with DAPI and examined using an Olympus BX63 fluorescence microscope (Tokyo, Japan).

### 2.7. Immunofluorescence Assay

Immunofluorescence analysis of hepatic Nrf2 was conducted as described previously [15]. Briefly, the cryostat section of liver tissue (8 *μ*M) was fixed in cooled acetone for 10 min at 4°C and then washed with PBS. After blocking the endogenous peroxidase with 5% goat serum for 20 min, the liver section was incubated with 1 : 100 rabbit anti-mouse Nrf2 antibody (Santa Cruz Biotechnology, Dallas, USA) at 4°C overnight and then incubated with 1 : 1000 Alexa Fluor® 568-labeled secondary antibody (Life Technologies, Carlsbad, CA, USA) in the dark at room temperature for 1 h. Nuclei were counterstained with DAPI for 10 min. The fluorescence was observed and photographed by an Olympus BX63 fluorescent microscope (Olympus).

### 2.8. RT-PCR Assay

Total RNA from the same lobe of liver tissue was extracted by a commercial RNAiso Plus kit according to the manufacturer's protocol (Takara, Tokyo, Japan). cDNA was synthesized by reverse transcription and amplified by PCR with the primers shown in Table 1 using PrimeScript RT Reagent kit (Takara). The sequence of primers was designed from the PrimerBank and synthesized by Invitrogen Life Technologies (Shanghai, China). PCR products were separated by agarose gel electrophoresis, stained with ethidium bromide, and visualized under UV light.

### 2.9. Western Blotting Assay

Total protein, cytosolic protein (exclusively for Nrf2), and nuclear protein (exclusively for Nrf2) from liver tissues were prepared. Total proteins were isolated from liver tissue by RIPA buffer with 1% phosphatase and protease inhibitors (Beyotime). The extraction and isolation of nuclear and cytosolic proteins were conducted by a Nuclear and Cytoplasmic Protein Extraction Kit (Beyotime). Protein concentration was quantified by using a Pierce BCA Kit (Thermo Fisher). An aliquot of 20 *μ*g total protein was loaded and separated on 10–15% SDS-PAGE and then, subsequently, electrophoretically transferred onto a polyvinylidene fluoride (PVDF) membrane. The transferred membrane was incubated with primary antibodies at 4°C overnight (Table 2) and then incubated with the corresponding secondary antibodies (1 : 1000) at room temperature for 1 h. The band was visualized by ECL Detection Reagent (GE Healthcare BioSciences, NJ, USA) in a FluorChem Imaging system (Cell Biosciences, Santa Clara, CA, USA).

### 2.10. Statistical Analysis

Data are presented as mean ± standard deviation (SD). To test the difference between groups, one-way analysis of variance (ANOVA) followed by Tukey's* post hoc *test was performed by using GraphPad Prism 6.0 software (San Diego, CA, USA). Statistical significance was accepted at the level of *p* < 0.05.

## 3. Results

### 3.1. Fatty Acid Composition in Liver Tissues

To measure the effect of* fat-1* expression on hepatic fatty acid profile, liver tissues from* fat-1* and WT mice were determined by GC-MS. Because* fat-1* gene can encode n-3 PUFA desaturase that allows converting n-6 PUFA to n-3 PUFA in* fat-1* mice, compared with WT/CCl_4_ group, liver tissues from* fat-1*/CCl_4_ group exhibited higher amounts of n-3 PUFA, including ALA (18:3n-3), EPA (20:5n-3), and DHA (22:6n-3), and lower levels of n-6 PUFA, mainly linoleic acid (LA, 18:2n-6) and arachidonic acid (AA, 20:4n-6), leading to a remarkable increase in total n-3 PUFA and decreases in total n-6 PUFA and n-6/n-3 ratio (Table 3). The level of total saturated fatty acids (SFA) in* fat-1*/CCl_4_ mice was significantly higher, and the level of total monounsaturated fatty acids (MUFAs) tended to be lower. These results demonstrated that the expression of* fat-1* gene greatly elevated n-3 PUFA levels in the liver, although both groups were fed the identical diet.

CCl_4_ exposure also greatly altered the fatty acid composition in the liver. Compared to WT control, the WT/CCl_4_ group showed decreased levels in SFA, mainly 14:0, 16:0, and 18:0, and increased levels in MUFA, mainly 16:1 and 18:1, leading to increased ratios of 16:1/16:0 and 18:1/18:0, the fatty acid desaturation index. These findings also suggest that CCl_4_ challenge may increase the activity or expression of stearoyl-CoA desaturase-1 (SCD-1).

### 3.2. Endogenous n-3 PUFA Ameliorates the Features of Acute CCl_4_-Induced Liver Injury

**Figure 1:**
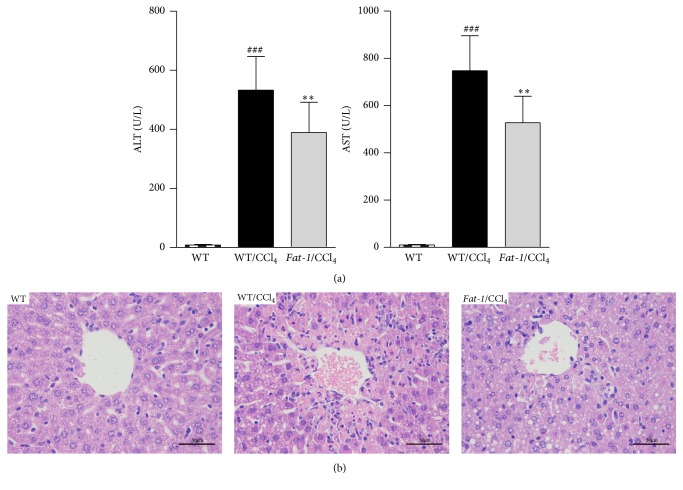
Endogenous n-3 PUFA alleviates CCl_4_-induced acute liver injury in* fat-1* mice. (a) Plasma levels of alanine aspartate transaminase (AST) and aminotransferase (ALT). (b) Representative hematoxylin and eosin (H&E) staining of liver tissue sections (magnification: 400x). Values represent the means ± SD (*n* = 10); ^###^ *p* < 0.001 versus WT group; ^*∗∗*^ *p* < 0.01 versus WT/CCl_4_ group.

Liver injury was evaluated by serum enzyme activities and hepatic histopathological changes. ALT and AST are released into the blood once the structural integrity of the hepatocyte was damaged; their levels are the most commonly used markers of liver injury [21]. As shown in Figure 1(a), after acute CCl_4_ challenge, the serum levels of ALT and AST in WT/CCl_4_ group increased 63 and 71 times, respectively, over those in WT group. However, these elevations were significantly blunted in* fat-1*/CCl_4_ group. The histological changes in the liver were evaluated by H&E staining (Figure 1(b)). WT group exhibited normal architecture with clear nuclear distribution. CCl_4_ induced histological changes including severely disrupted hepatic architecture and extensive hepatocellular necrosis around the blood vessels in WT/CCl_4_ group, which was reduced in* fat-1*/CCl_4_ group.

### 3.3. Endogenous n-3 PUFA Reduces CCl_4_-Induced Oxidative Stress in the Liver

Oxidative stress is characterized as a redox imbalance between prooxidants and endogenous antioxidants, including nonenzymatic antioxidants (e.g., GSH) and enzymatic antioxidants (e.g., SOD, CAT, and GSH-Px) [22]. MDA is an end product of lipid peroxidation (LPO) and has been widely used as a marker of oxidative stress [22]. As shown in Table 4, CCl_4_ exposure induced a remarkable increase of hepatic MDA production by 87.7% (2.52 ± 0.34 versus 4.73 ± 0.52, *p* < 0.01), and a remarkable decrease in hepatic lipid peroxidation was observed in* fat-1*/CCl_4_ group. Conversely, CCl_4_ challenge depleted endogenous enzymatic and nonenzymatic antioxidants which can protect hepatocytes against oxidative stress, as it is indicated that the activities of SOD, CAT, and GSH-Px and GSH level in WT/CCl_4_ group were significantly reduced to 70.5%, 55.7%, 59.0%, and 68.4% of those of WT group, respectively. This depletion of endogenous antioxidants was markedly ameliorated in* fat-1*/CCl_4_ group. As a radical scavenger, GSH can be oxidized to GSSG under oxidative stress. GSSG is also reduced back to GSH by glutathione reductase (GR). Hence, GSH/GSSG has been also used as a marker of oxidative stress [23]. CCl_4_ exposure significantly increased the GSSG level and decreased GR activity in the liver, leading to a great decrease in GSH/GSSG ratio; these changes were remarkably ameliorated in* fat-1* mice.

### 3.4. Endogenous n-3 PUFA Upregulates Antioxidant Enzymes via Nuclear Translocation of Nrf2

**Figure 2:**
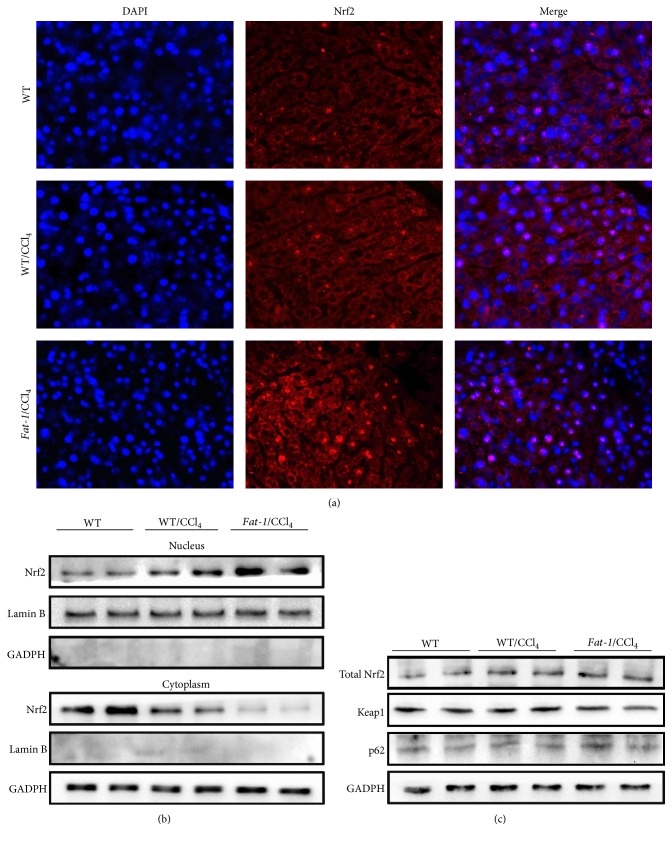
Endogenous n-3 PUFA induces nuclear translocation of Nrf2. (a) Immunofluorescence staining of Nrf2. (b) Western blot analysis of Nrf2 in the nucleus and cytoplasm. (c) Western blot analysis of total Nrf2, keap1, and p62 in liver tissue.

To understand the underlying molecular mechanisms for the protective effects of endogenous n-3 PUFA against oxidative stress triggered by CCl_4_, the activation of Nrf2, a main transcription factor regulating antioxidant responses in the liver, was examined by immunofluorescence assay and immunoblot analysis. As shown in Figure 2(a), the significant nuclear translocation of Nrf2 was detected in* fat-1*/CCl_4_ group, compared to WT/CCl_4_ group, which was in accordance with the results of immunoblot analysis. Endogenous n-3 PUFA in* fat-1 *mice greatly decreased the protein expression of Nrf2 in the cytoplasm but increased Nrf2 expression in the nucleus, without changing the level of total Nrf2 expression in the liver (Figure 2(b)). In addition, the Kelch-like ECH-associated protein-1 (Keap1), a repressor protein, and p62, a substrate adaptor sequestosome-1 protein that competes with Nrf2 for binding to Keap1, were examined in the liver by western blot. As shown in Figure 2(c), the lower protein expression of Keap1 and higher expression of p62 were detected in* fat-1*/CCl_4_ mice compared to WT/CCl_4_ group.

**Figure 3:**
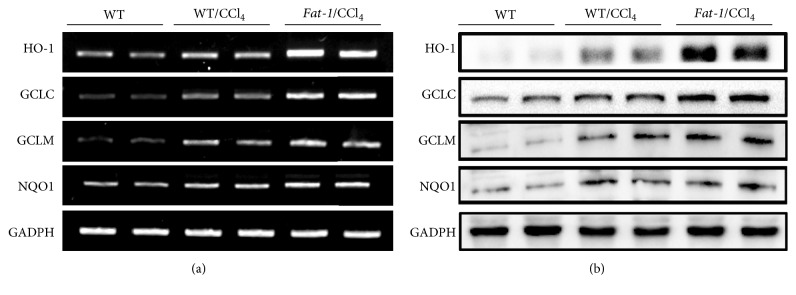
Endogenous n-3 PUFA upregulates mRNA (a) and protein (b) expressions of Nrf2 target genes, including HO-1, GCLC, GCLM, and NQO1.

Nrf2-regulated genes, such as HO-1, GCLC, GCLM, and NQO1, were also examined. As shown in Figure 3, both mRNA and protein levels of HO-1, GCLC, GCLM, and NQO1 in the liver were obviously upregulated in* fat-1*/CCl_4_ group compared to WT/CCl_4_ group. Interestingly, CCl_4_ challenge notably promoted nuclear translocation of Nrf2, elevated Nrf2 expression in the nucleus, and increased the expression of its downstream genes (Figures 2 and 3), which were consistent with the previous studies [3, 4]. A most plausible explanation is the adaptive cytoprotective reaction of organisms in response to oxidative stimuli. These results demonstrate that the protection of endogenous n-3 PUFA against CCl_4_-induced liver damage is correlated with ameliorating oxidative stress in the liver via activating Nrf2 and upregulating its downstream genes.

### 3.5. Endogenous n-3 PUFA Reduces Hepatocyte Apoptosis via Regulating MAPK Signal Pathway

**Figure 4:**
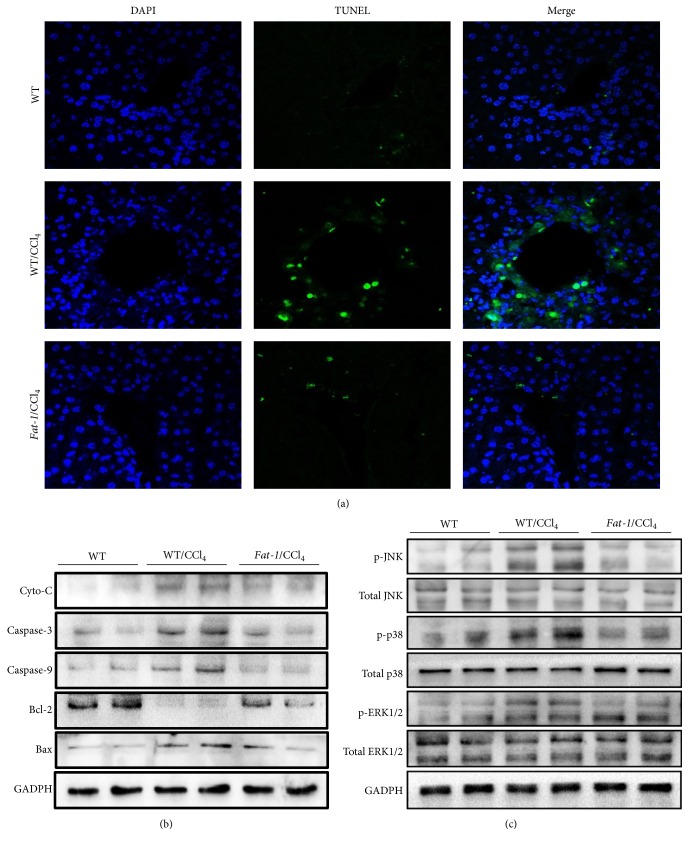
Endogenous n-3 PUFA protects against CCl_4_-induced hepatocyte apoptosis in* fat-1* mice via regulating MAPK signaling pathway. (a) Representative images of TUNEL stained liver sections (magnification: 200x): green fluorescence indicates the positive cells, and cellular nucleus is labeled by staining DAPI with blue fluorescence. (b) Western blot analysis of apoptosis-related proteins, including cytochrome C, caspase-3, caspase-9, Bcl-2, and Bax. (c) Western blot analysis of total and phosphorylated protein expression of JNK, p38, and ERK.

As cell apoptosis directly reflects the extent of liver injury caused by CCl_4_, a TUNEL assay was conducted to estimate the regulation of cell apoptosis by endogenous n-3 PUFA. As shown in Figure 4(a), after 24 h of CCl_4_ challenge, the number of TUNEL-positive cells in the liver section was significantly increased over the WT control group. In* fat-1/*CCl_4_ group, this increase in apoptotic cells was significantly decreased. In addition, during the CCl_4_-induced liver injury, there was a cascade of apoptosis-related molecular events [24, 25]. After CCl_4_ challenge, the protein expressions of the proapoptotic proteins, including cytochrome C, caspase-3, caspase-9, and Bax, were obviously increased in liver tissues from WT mice, while the levels of the antiapoptotic factor Bcl-2 was significantly decreased. In agreement with the reduction of TUNEL-positive cells, compared with WT/CCl_4_ mice, the* fat-1*/CCl_4_ mice showed decreased levels of the aforementioned proapoptotic proteins and increased level of Bax in the liver, as indicated by western blot analyses (Figure 4(b)). These results indicate that endogenous n-3 PUFA effectively prevents CCl_4_-induced DNA fragmentation.

To understand the underlying molecular mechanisms for the inhibitory effects of endogenous n-3 PUFA on CCl_4_-induced hepatocyte apoptosis, ERK, JNK, and p38, the major components in mitogen-activated protein kinase (MAPK) pathways, which are critical regulators of cell proliferation and death in response to diverse stresses, were examined by immunoblotting. Oxidative stress in the liver activates MAPK after CCl_4_ challenge and results in activation of JNK, p38, and ERK [25]. As shown in Figure 4(c), CCl_4_ exposure obviously increased the phosphorylated protein levels of JNK, p38, and ERK1/2, without changing their total expressions in liver tissues from WT mice. These increases in phosphorylated kinases were all downregulated by endogenous n-3 PUFA in* fat-1* mice. Thus, protective effects of endogenous n-3 PUFA against CCl_4_-induced hepatocyte apoptosis are associated with suppressing MAPK pathways.

## 4. Discussion

In this study, we used* fat-1* transgenic mice to investigate the role of endogenous n-3 PUFA in CCl_4_-caused acute liver damage. We demonstrate that CCl_4_ challenge caused severe liver injury in WT mice, as illustrated by markedly elevated serum activities of AST and ALT, oxidative stress, and hepatocyte apoptosis. Those pathological alterations were remarkably relieved in* fat-1* mice after CCl_4_ challenge, which was associated with activating Nrf2 and regulating MAPK signal pathway.

The* fat-1* transgenic mice have been widely used as a novel tool for investigating the benefits of long chain n-3 PUFAs and the mechanisms underlying their actions [26].* Fat-1* transgenic mice, carrying a* fat-1* gene from* C. elegans*, encode a desaturase that can convert n-3 to n-6 PUFA, resulting in abundant n-3 PUFA, without changing total PUFA in their organs and tissues (Table 3).* Fat-1* mice and WT littermates endogenously generate distinct fatty acid profiles in the liver while feeding them the same diet rich in n-6 PUFA. Thus, several variables arising from different diets, such as flavor, oxidation degree, and unwanted components of fat used, may be well avoided [15]. As expected, in this study, liver tissues from* fat-1*/CCl_4_ group exhibited higher amounts of n-3 PUFA, particularly EPA and DHA, and lower level of n-6 PUFA, leading to a remarkable increase in total n-3 PUFA in the liver, compared to WT/CCl_4_ group. As a well-characterized animal model, the* fat-1* mice were studied to examine the impacts of endogenous n-3 PUFA on CCl_4_-induced acute liver injury.

Oxidative stress is critical during the pathogenesis of CCl_4_-induced acute liver injury [27]. CCl_4_ challenge produces highly reactive species and increases cellular production of ROS and MDA, leading to oxidative stress in tissues, especially the liver where it is primarily metabolized [6]. Additionally, CCl_4_-induced oxidative stress also depletes antioxidant defense system, including endogenous nonenzymatic (e.g., GSH) and enzymatic (e.g., SOD, CAT, GSH-Px, and GR) antioxidants. GSH has been considered to be the first line of defense against free radicals. It was documented that GSH is an important antioxidant in eliminating toxic free radicals and reactive toxic CCl_4_ metabolites [28, 29]. The sulfhydryl residues of GSH molecule are easily oxidized to GSSG, which can be reduced back to GSH by GR [30]. Thus, GSH/GSSG ratio serves as a reliable marker to evaluate the redox status and potential of oxidative stress [30]. In our study, CCl_4 _challenge increased hepatic MDA and GSSG and the ratio of GSH/GSSG and depleted GSH, SOD, CAT, GSH-Px, and GR in livers of WT mice, which was ameliorated in* fat-1* mice after CCl_4_ treatment (Table 4). To examine how n-3 PUFA improves the antioxidant defense system, the nuclear translocation of Nrf2 and the expressions of Nrf2 target genes in the liver were evaluated. Nrf2 acts as a transcription factor which plays a key role in regulating the expression of antioxidant proteins in response to oxidative stress [31]. Under physiological condition, Nrf2 is attached to Keap1, a specific repressor, in the cytoplasm, which promotes Nrf2 degradation by the ubiquitin proteasome pathway [32]. In the presence of ROS, Nrf2 degradation ceases, while stabilized Nrf2 translocates into the nucleus, which triggers the expression of a series of antioxidants, including HO-1, GCLC, GCLM, and NQO1, through antioxidant response element (ARE). HO-1 is a strong antioxidant with antiapoptotic and anti-inflammatory effects in the liver. GCLC and GCLM are key rate-limiting enzymes in GSH biosynthesis [33]. p62 is a substrate adaptor sequestosome-1 protein that modulates the Nrf2-Keap1 signaling pathway by competing with Nrf2 for binding to Keap1. In this study, the significant nuclear translocation of Nrf2 was observed in* fat-1*/CCl_4_ group, as evidenced by immunofluorescence assay and immunoblot analysis (Figure 2). Additionally,* fat-1*/CCl_4_ mice showed lower protein expression of Keap1 and higher expressions of p62, HO-1, GCLC, GCLM, and NQO1 in the liver, when compared to WT/CCl_4_ mice (Figure 3). These results suggest that the protective effects of endogenous n-3 PUFA against CCl_4_-caused acute liver damage might be attributable to reducing oxidative stress via the activation of Nrf2-keap1 pathway.

The induction of hepatocyte apoptosis has been well studied in acute liver injury induced by CCl_4_ exposure [34]. Our data demonstrated that CCl_4_ challenge markedly induced hepatocyte apoptosis in WT mice (Figure 4(a)), which was significantly reduced in* fat-1* mice after CCl_4_ exposure. The mitochondrial apoptotic pathway was considered to be involved in various types of cellular stress [35]. Oxidative stress causes elevated cytochrome C in the cytoplasm which is released from the mitochondria, which consequently induces the activation of caspase cascades, including caspase-3 and caspase-9. The mitochondrial apoptotic pathway is also regulated by several apoptosis-related factors, such as Bax and Bcl-2 [36]. The ratio of proapoptotic protein Bax to antiapoptotic protein Bcl-2 is critical for cell death or survival. Increased Bax/Bcl-2 ratio leads to cytochrome C release, caspase-3 activation, and eventually apoptosis [37]. Our data revealed that endogenous n-3 PUFA significantly inhibited the upregulation of cytochromeC, caspase-3, caspase-9, and Bax and normalized the downregulation of Bcl-2 expression induced by CCl_4_ (Figure 4(b)), which was well consistent with TUNEL staining results.

The MAPK family members, including JNK, p38, and JNK, are crucial for the regulation of cell proliferation, differentiation, apoptosis, and cellular responses to oxidative stress [38, 39]. Activated MAPKs can inactivate Bcl-2 by phosphorylation, activate caspase-9, and regulate the release of cytochrome C from the mitochondria [40]. Previous studies revealed that suppressing protein expressions of the phosphorylated MAPK members contributed to the inhibition of CCl_4_-induced apoptosis [38, 39]. In this study, the upregulation of phosphorylated JNK, p38, and JNK induced by CCl_4_ challenge was attenuated in the livers of* fat-1* mice. These findings suggest that reducing hepatocyte apoptosis via suppressing MAPK pathway might also contribute to the inhibitory function of n-3 PUFA on CCl_4_-induced liver injury.

**Figure 5:**
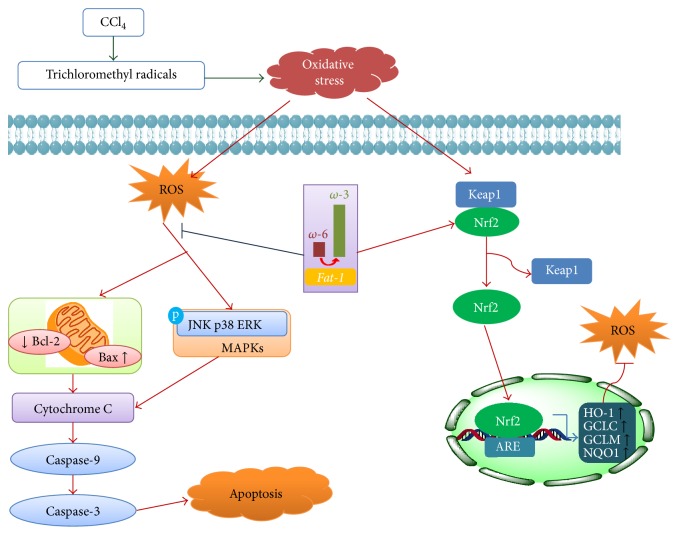
A schematic diagram of the potential mechanisms that underlie the protective effects of n-3 PUFA against CCl_4_-induced acute liver injury.

In conclusion, endogenous n-3 PUFA effectively ameliorated CCl_4_-induced acute liver injury, and this protective effect might be associated with ameliorating oxidative stress via Nrf2 activation and reducing apoptosis via suppression of MAPK pathway, as illustrated in Figure 5. Our findings suggest that dietary supplement with n-3 PUFA may be beneficial for the prevention of liver injury.

## Figures and Tables

**Table 1:** The primer sequences used in quantitative reverse transcription PCR analysis.

Gene	Full name	GenBank accession number	Primer sequences (forward/reverse)
*HO-1*	Heme oxygenase-1	NM_010442	5′-AAGCCGAGAATGCTGAGTTCA-3′
			5′-GCCGTGTAGATATGGTACAAGGA-3′
*GCLC*	Glutamate cysteine ligase catalytic subunit	NM_010295	5′-GGGGTGACGAGGTGGAGTA-3′
			5′-GTTGGGGTTTGTCCTCTCCC-3′
*GCLM*	Glutamate cysteine ligase modifier subunit	NM_008129	5′-AGGAGCTTCGGGACTGTATCC-3′
			5′-GGGACATGGTGCATTCCAAAA-3′
*NQO1*	Quinone oxidoreductase-1	NM_008706	5′-AGGATGGGAGGTACTCGAATC-3′
			5′-AGGCGTCCTTCCTTATATGCTA-3′
*GADPH*	Glyceraldehyde-3-phosphate dehydrogenase	NM_008085	5′-TGGATTTGGACGCATTGGTC-3′
			5′-TTTGCACTGGTACGTGTTGAT-3′

**Table 2:** Primary antibodies used in immunoblot analysis.

Primary antibody	Full name	Source	Dilution	Company
Bax	BCL2-associated X protein	Rabbit	1 : 1000	Cell Signaling Technology
Bcl-2	B-cell lymphoma-2	Rabbit	1 : 1000	Cell Signaling Technology
Caspase-3	Cysteinyl aspartate specific proteinase-3	Rabbit	1 : 1000	Cell Signaling Technology
Caspase-9	Cysteinyl aspartate specific proteinase-9	Rabbit	1 : 1000	Cell Signaling Technology
CYP2E1	Cytochrome P4502E1	Rabbit	1 : 500	Abcam
Cyto-C	Cytochrome C	Rabbit	1 : 1000	Cell Signaling Technology
ERK	Extracellular signal-regulated protein kinase	Rabbit	1 : 1000	Cell Signaling Technology
GADPH	Glyceraldehyde-3-phosphate dehydrogenase	Rabbit	1 : 1000	Cell Signaling Technology
GCLC	Glutamate cysteine ligase catalytic subunit	Rabbit	1 : 1000	Abcam
GCLM	Glutamate cysteine ligase modifier subunit	Rabbit	1 : 500	Santa Cruz Biotechnology
HO-1	Heme oxygenase-1	Rabbit	1 : 1000	Abcam
JNK	c-Jun N-terminal kinase	Rabbit	1 : 1000	Cell Signaling Technology
Keap1	Kelch-like ECH-associated protein-1	Rabbit	1 : 1000	Cell Signaling Technology
Lamin B	Lamin B	Rabbit	1 : 500	Cell Signaling Technology
Nrf2	Nuclear factor erythroid 2-related factor-2	Rabbit	1 : 1000	Santa Cruz Biotechnology
NQO1	NAD(P)H:quinone oxidoreductase-1	Rabbit	1 : 1000	Santa Cruz Biotechnology
p38	p38 mitogen-activated protein kinase	Rabbit	1 : 1000	Cell Signaling Technology
p62	Nucleoporin p62	Rabbit	1 : 1000	Cell Signaling Technology
p-ERK	Phosphorylated extracellular signal-regulated protein kinase	Rabbit	1 : 1000	Cell Signaling Technology
p-JNK	Phosphorylated c-Jun N-terminal kinase	Rabbit	1 : 1000	Cell Signaling Technology
p-p38	Phosphorylated p38 mitogen-activated protein kinase	Rabbit	1 : 1000	Cell Signaling Technology

**Table 3:** Fatty acid composition (%) of liver tissues.

Fatty acids			% of total fatty acids		
Common name	Symbol		WT	WT/CCl_4_	*Fat-1*/CCl_4_
Lauric acid	12:0		0.27 ± 0.22	0.23 ± 0.15	0.26 ± 0.22
Myristic acid	14:0		0.84 ± 0.28	0.60 ± 0.13^#^	0.76 ± 0.11^*∗∗*^
Palmitic acid	16:0		28.5 ± 4.3	24.4 ± 1.6^#^	26.7 ± 3.8
Palmitoleic acid	16:1		1.16 ± 0.43	1.59 ± 0.42^#^	1.32 ± 0.38^*∗∗*^
Stearic acid	18:0		23.0 ± 7.4	14.5 ± 2.9^##^	19.5 ± 5.4^*∗*^
Oleic acid	18:1		15.1 ± 5.0	20.8 ± 2.3^##^	17.7 ± 3.1^*∗*^
Linolenic acid	18:2,6		17.1 ± 5.6	24.6 ± 2.3^##^	21.3 ± 4.2^*∗*^
*γ*-Linolenic acid	18:3,6		0.41 ± 0.20	0.35 ± 0.10	0.28 ± 0.16
*α*-Linolenic acid	18:3,3		0.27 ± 0.05	0.22 ± 0.05	0.36 ± 0.07^*∗∗*^
Arachidic acid	20:0		0.45 ± 0.18	0.23 ± 0.08^##^	0.41 ± 0.12^*∗∗*^
Eicosenoic acid	20:1		0.26 ± 0.06	0.26 ± 0.03	0.25 ± 0.05
Dihomo-*γ*-linoleic acid	20:3,6		0.43 ± 0.14	0.60 ± 0.10^##^	0.43 ± 0.08^*∗∗*^
Arachidonic acid	20:4,6		9.08 ± 1.48	8.21 ± 1.86	4.73 ± 1.29^*∗∗*^
Eicosapentaenoic acid	20:5,3		0.03 ± 0.02	0.04 ± 0.03	0.42 ± 0.16^*∗∗*^
Behenic acid	22:0		0.17 ± 0.05	0.15 ± 0.05	0.17 ± 0.01
Erucic acid	22:1		0.20 ± 0.06	0.18 ± 0.11	0.29 ± 0.08^*∗*^
Docosadienoic acid	22:2,6		0.25 ± 0.10	0.10 ± 0.11^##^	0.02 ± 0.01^*∗*^
Docosatetraenoic acid	22:4,6		0.28 ± 0.16	0.45 ± 0.12^##^	0.22 ± 0.10^*∗∗*^
Docosapentaenoic acid	22:5,3		0.04 ± 0.03	0.07 ± 0.06	0.15 ± 0.21
Lignoceric acid	24:0		0.02 ± 0.01	0.02 ± 0.02	0.01 ± 0.01
Docosahexaenoic acid	22:6,3		1.67 ± 0.39	1.94 ± 0.33	4.16 ± 1.17^*∗∗*^
Nervonic acid	24:1		0.09 ± 0.04	0.20 ± 0.09^##^	0.13 ± 0.09
SFAs			53.5 ± 11.9	40.4 ± 4.1^##^	48.1 ± 9.1^*∗∗*^
MUFAs			16.9 ± 5.5	23.1 ± 2.7^##^	19.8 ± 3.6^*∗*^
n-3 PUFAs			2.04 ± 0.38	2.30 ± 0.33	5.1 ± 1.4^*∗∗*^
n-6 PUFAs			27.5 ± 6.5	34.3 ± 2.8	27.0 ± 5.6^*∗*^
Total PUFAs			29.6 ± 6.8	36.6 ± 3.0	32.2 ± 6.8
n-6/n-3 PUFAs			13.4 ± 1.5	15.1 ± 1.7	5.40 ± 0.75^*∗∗*^
16:1/16:0			0.04 ± 0.02	0.07 ± 0.02^##^	0.05 ± 0.02
18:1/18:0			0.80 ± 0.52	1.51 ± 0.42^##^	1.01 ± 0.41^*∗∗*^

Values are expressed as the means ± SD (*n* = 10); ^#^ *p* < 0.05 and ^##^ *p* < 0.01 versus WT group; ^*∗*^ *p* < 0.05 and ^*∗∗*^ *p* < 0.01 versus WT/CCl_4_ group. SFA: saturated fatty acid; MUFA: monounsaturated fatty acid; PUFA: polyunsaturated fatty acid.

**Table 4:** Effects of endogenous omega-3 fatty acids on oxidative stress parameters in the liver.

Parameters	WT	WT/CCl_4_	*Fat-1*/CCl_4_
MDA (nmol/mg protein)	2.52 ± 0.34	4.73 ± 0.52^##^	3.99 ± 0.54^*∗∗*^
SOD (U/mg protein)	303.8 ± 33.8	214.3 ± 32.8^#^	261.8 ± 44.2^*∗∗*^
CAT (U/mg protein)	13.5 ± 1.9	7.52 ± 2.26^#^	9.97 ± 2.10^*∗*^
GSH-Px (U/mg protein)	975.6 ± 317.2	565.4 ± 199.1^##^	770.1 ± 283.4^*∗∗*^
GR (U/g protein)	10.6 ± 1.7	6.22 ± 1.77^##^	7.92 ± 1.72^*∗*^
GSH (mg/g protein)	11.6 ± 1.8	7.96 ± 0.89^##^	9.27 ± 1.93^*∗∗*^
GSSG (mg/g protein)	1.26 ± 0.36	2.29 ± 0.56^##^	1.72 ± 0.40^*∗*^
GSH/GSSG (fold)	9.72 ± 2.27	3.62 ± 0.89^##^	5.46 ± 0.94^*∗∗*^

Data are expressed as mean ± SD (*n* = 10). ^#^ *p* < 0.05 and ^##^ *p* < 0.01 versus WT group; ^*∗*^ *p* < 0.05 and ^*∗∗*^ *p* < 0.01 versus WT/CCl_4_ group.

## Abbreviations

ALT: Alanine transaminase
ARE: Antioxidant response element
AST: Aspartate transaminase
Bax: Bcl-2-associated X protein
Bcl-2: B-cell lymphoma-2
CAT: Catalase
Caspase-3: Cysteinyl aspartate specific proteinase-3
Caspase-9: Cysteinyl aspartate specific proteinase-9
CCl_4_: Carbon tetrachloride
CYP2E1: Cytochrome P4502E1
Cyto-C: Cytochrome C
DAPI: 4,6-Diamidino-2-phenylindole
ERK: Extracellular signal-regulated kinase
FAME: Fatty acid methyl esters
GADPH: Glyceraldehyde-3-phosphate dehydrogenase
GCLC: Glutamate cysteine ligase catalytic subunit
GCLM: Glutamate cysteine ligase modifier subunit
GSH: Reduced glutathione
GSH-Px: Glutathione peroxidase
GSSG: Oxidized glutathione
H&E: Hematoxylin and eosin
HO-1: Heme oxygenase-1
JNK: c-Jun N-terminal kinase
Keap1: Kelch-like ECH-associated protein-1
MAPK: Mitogen-activated protein kinase
MDA: Malondialdehyde
MUFA(s): Monounsaturated fatty acid(s)
Nrf2: Nuclear factor-erythroid 2-related factor-2
NQO1: NAD(P)H:quinone oxidoreductase-1
PUFA(s): Polyunsaturated fatty acid(s)
p62: Nucleoporin p62
ROS: Reactive oxygen species
SFA(s): Saturated fatty acid(s)
SOD: Superoxide dismutase
TUNEL: Terminal deoxynucleotidyl transferase-mediated deoxyuridine 5-triphosphate nick end labeling
WT: Wild-type.
